# CTRP3 Protects against High Glucose-Induced Cell Injury in Human Umbilical Vein Endothelial Cells

**DOI:** 10.1155/2019/7405602

**Published:** 2019-07-24

**Authors:** Fang Wang, Linlin Zhao, Yingguang Shan, Ran Li, Guijun Qin

**Affiliations:** ^1^Department of Endocrinology, The First Affiliated Hospital of Zhengzhou University, Zhengzhou, China; ^2^Department of Cardiology, The First Affiliated Hospital of Zhengzhou University, Zhengzhou, China

## Abstract

**Aims:**

Inflammation was closely associated with diabetes-related endothelial dysfunction. C1q/tumor necrosis factor-related protein 3 (CTRP3) is a member of the CTRP family and can provide cardioprotection in many cardiovascular diseases via suppressing the production of inflammatory factors. However, the role of CTRP3 in high glucose- (HG-) related endothelial dysfunction remains unclear. This study evaluates the effects of CTRP3 on HG-induced cell inflammation and apoptosis.

**Materials and Methods:**

To prevent high glucose-induced cell injury, human umbilical vein endothelial cells (HUVECs) were pretreated with recombinant CTRP3 for 1 hour followed by normal glucose (5.5 mmol/l) or high glucose (33 mmol/l) treatment. After that, cell apoptosis and inflammatory factors were determined.

**Results:**

Our results demonstrated that CTRP3 mRNA and protein expression were significantly decreased after HG exposure in HUVECs. Recombinant human CTRP3 inhibited HG-induced accumulation of inflammatory factors and cell loss in HUVECs. CTRP3 treatment also increased the phosphorylation levels of protein kinase B (AKT/PKB) and the mammalian target of rapamycin (mTOR) in HUVECs. CTRP3 lost its inhibitory effects on HG-induced cell inflammation and apoptosis after AKT inhibition. Knockdown of endogenous CTRP3 in HUVECs resulted in increased inflammation and decreased cell viability in vitro.

**Conclusions:**

Taken together, these findings indicated that CTRP3 treatment blocked the accumulation of inflammatory factors and cell loss in HUVECs after HG exposure through the activation of AKT-mTOR signaling pathway. Thus, CTRP3 may be a potential therapeutic drug for the prevention of diabetes-related endothelial dysfunction.

## 1. Introduction

Hyperglycemia is one of the major causes of vascular complications in patients with diabetes [1, 2]. Diabetes-related endothelial dysfunction contributed to the development of diabetic vascular complications [3]. Endothelial dysfunction could be observed in the initial stage of diabetes [4]. Sustained high glucose (HG) induced profound production of inflammatory factors in endothelial cells, thus resulting in the death of the endothelium and apoptosis [5, 6]. Short-term high glucose exposure increased monocyte chemotactic protein 1 (MCP-1) expression in human aortic endothelial cells to enhance monocyte-endothelial cell adhesion [7]. Therefore, to prevent diabetes-related endothelial dysfunction, it is imperative to find molecules that could inhibit HG-induced inflammation and cell apoptosis.

C1q/tumor necrosis factor-related proteins (CTRPs) are a highly conserved family of adiponectin paralogs. CTRP3, also called CORS-26, has been reported to be highly expressed in cardiac samples [8, 9]. CTRP3 is composed of four domains, including an N-terminal domain, a short variable domain, a collagen-like domain, and a C-terminal globular domain [10]. Globular CTRP3 (lacking parts at the N-terminal domain) was used frequently to investigate the biological functions of CTRP3 [9, 11]. Ma et al. found that CTRP3 improved cardiac dysfunction and prevented diabetes-related cardiac injury in rats [9]. Previous studies have found that CTRP3 suppressed cardiomyocyte apoptosis and prevented cardiac fibrosis in ischemic mouse hearts [12]. CTRP3 also attenuated doxorubicin-induced cardiac death and reduced sepsis-induced myocardial dysfunction in mice [11, 13]. Notably, CTRP3 could act as a novel endogenous antagonist of LPS and prevent LPS-related inflammation [14]. However, there is still a paucity of data regarding the effects of CTRP3 on HG-induced endothelial dysfunction.

Therefore, we conducted the present study to explore the potential effects of CTRP3 on diabetes-related endothelial dysfunction in human umbilical vein endothelial cells (HUVECs). We found that CTRP3 was downregulated in HUVECs after high glucose treatment and that CTRP3 administration reduced high glucose-induced cell inflammation and cell death via activating the protein kinase B (AKT/PKB) signaling pathway.

## 2. Methods and Materials

### 2.1. Reagents

Recombinant CTRP3 globular form (human) was obtained from Aviscera Bioscience (CA, USA). The following primary antibodies were purchased from Abcam (Boston, MA, USA): anti-CTRP3 antibody (1 : 1000, ab36870), anti-glyceraldehyde 3-phosphate dehydrogenase (GAPDH, 1 : 1000, ab181602), anti-nuclear factor-*κ*B (NF-*κ*B, phospho S536) antibody (1 : 1000, ab86299), anti-NF-*κ*B p65 antibody (1 : 1000, ab16502), anti-proliferating cell nuclear antigen (PCNA, 1 : 1000, ab92552), anti-Bax (ab32503, 1 : 1000), anti-B-cell lymphoma 2 (Bcl-2, ab196495, 1 : 1000), anti-AKT (phospho T308, 1 : 1000, ab38449), anti-AKT (1 : 1000, ab8805), anti-mammalian target of rapamycin (p-mTOR, 1 : 1000, ab137133, phospho S2481), and anti-mTOR (1 : 1000, ab2732). A specific AKT1/2 kinase inhibitor was obtained from Sigma-Aldrich (No. A6730, St. Louis, MO, USA). Isolation of nuclear proteins was performed using a kit from Beyotime Biotechnology (No. P0028, Beijing, China).

### 2.2. Cell Culture and Treatment

HUVECs were obtained from the American Type Culture Collection (Manassas, VA, USA). This cell line was grown in Dulbecco's modified Eagle's medium (DMEM) supplemented with 10% fetal bovine serum at 37°C in a 5% CO_2_ humidified atmosphere. To induce high glucose-induced cell injury, cells were cultured in DMEM supplemented with 30 mmol/l glucose. The cells in the control group were cultured in the presence of 5 mmol/l glucose. To investigate the protective role of CTRP3, HUVECs were pretreated with recombinant CTRP3 (3 *μ*g/ml) for 1 hour followed by normal glucose (5.5 mmol/l) or high glucose (33 mmol/l) treatment. The dose of CTRP3 was determined according to a previous study [9]. To confirm the requirement of AKT activation in CTRP3-provided protection, HUVECs were incubated with AKT1/2 kinase inhibitor (1 *μ*mol/l). To determine the role of endogenous CTRP3, cells were incubated with siCTRP3 (50 mmol/l) for 24 h to knock down the expression of CTRP3 and siRNA was used as a negative control.

### 2.3. Western Blot

HUVECs were lysed in RIPA buffer containing phenylmethylsulfonyl fluoride (PMSF, 1 mmol/l) and phosphatase inhibitors (0.01 mmol/l). The protein concentration was determined by a bicinchoninic acid (BCA) protein assay (Beyotime Biotechnology, China). For each detection, proteins (20 *μ*g) were separated by 6% (mTOR) or 10% SDS-PAGE and electrotransferred onto PVDF membranes (Millipore, Billerica, MA, USA). After blocking with 5% nonfat milk, the membranes were reacted with primary antibodies (4°C overnight) and then reacted with the secondary antibody (room temperature for 1 h). After that, the membranes were examined using the enhanced chemiluminescence reagents (Amersham, Buckinghamshire, UK). The intensity of the band was counted using the ImageJ software.

### 2.4. Quantitative Real-Time Polymerase Chain Reaction Assay

Total RNA from the HUEVCs was extracted using TRIzol reagent (Invitrogen, United States) [15]. Then, the purity and concentration of the RNA were detected. After that, total RNA was reverse-transcribed to cDNA using the PrimeScript™ RT Reagent Kit with gDNA Eraser. The quantification of real-time PCR was performed using an ABI Prism 7700 Real-Time PCR System (Applied Biosystems, Foster City, CA) with the SYBR Green PCR Kit (Takara). GAPDH was used as the reference control.

### 2.5. TUNEL Staining

To detect cell apoptosis, HUVECs were seeded into a 24-well plate at an intensity of 5 × 10^4^ cells/cm^2^. After HG exposure for 72 hours, TUNEL assay was also conducted using a transferase-mediated dUTP nick-end labeling (TUNEL) kit (Roche, Germany) according to the manufacturer's instructions.

### 2.6. Cell Viability Assay

HUVECs were seeded into 96-well plates at an intensity of 5 × 10^4^ cells/cm^2^. After HG exposure for 72 hours, cell viability was measured using a Cell Counting Kit-8 (CCK-8, Dojindo Laboratories, Kumamoto, Japan). The optical density (OD) values were evaluated at the wavelength of 550 nm using a microplate reader.

### 2.7. Statistical Analysis

All the data were expressed as the mean ± standard deviation (SD). Statistical analysis among multiple groups was carried out using one-way analysis of variance followed by Tukey post hoc. We used the Student *t*-test to compare significance between two groups.

## 3. Result

### 3.1. CTRP3 Was Downregulated by High Glucose in HUVECs

**Figure 1:**
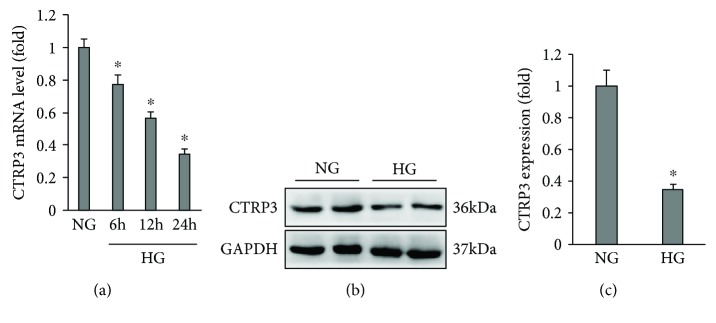
CTRP3 expression in HUVECs exposed to high glucose. (a) CTRP3 mRNA levels in HUVECs exposed to HG for indicated different times (*n* = 5). (b) Protein levels of CTRP3 expressed in HUVECs exposed to HG for 24 hours (*n* = 5). Data are expressed as mean ± SD of five independent experiments. ^∗^*P* < 0.05 when compared to the NG group.

To evaluate the role of CTRP3 in high glucose-induced endothelial dysfunction, we first detected CTRP3 expression in HUVECs. Using quantitative real-time PCR assay, we found that CTRP3 mRNA gradually decreased at different time points (6, 12, and 24 h) at a glucose concentration of 33 mmol/l in HUVECs (Figure 1(a)). Similarly, western blot analysis showed that CTRP3 protein expression was also decreased by high glucose exposure for 24 h in HUVECs (Figure 1(b)).

### 3.2. CTRP3 Treatment Inhibited the Accumulation of Inflammatory Factors in HUVECs after HG Exposure

**Figure 2:**
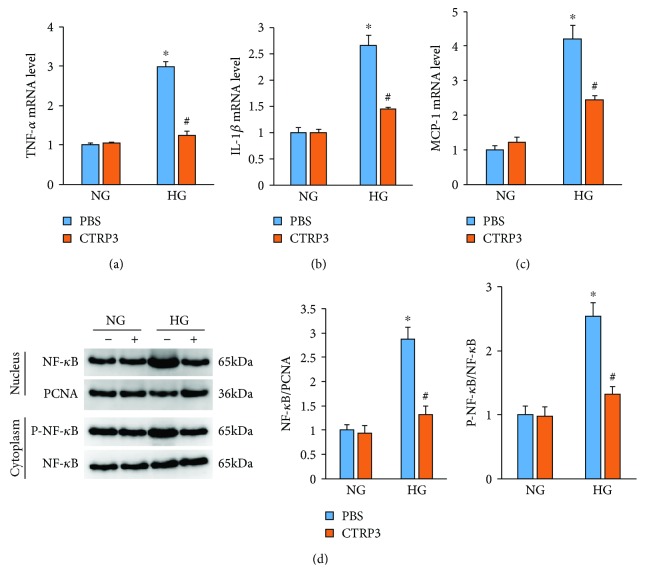
CTRP3 inhibits cell inflammation level in HUVECs induced by high glucose. (a–c) The mRNA expression of inflammatory factors in HUVECs exposed to high glucose (*n* = 5). (d) The protein expression of NF-*κ*B was detected by western blot analysis (*n* = 5). Data are expressed as mean ± SD of five independent experiments. ^∗^*P* < 0.05 when compared to the NG group; ^#^*P* < 0.05 when compared to the HG group.

To examine the effect of CTRP3 on the inflammation induced by HG, HUVECs were subjected to recombinant human CTRP3 globular form for 24 h. HG stimulation increased the expression of inflammatory factors, such as tumor necrosis factor-*α* (TNF-*α*), interleukin-1*β* (IL-1*β*), and MCP-1. However, these pathological elevations were suppressed by the treatment of CTRP3 (Figures 2(a)–2(c)). Nuclear NF-*κ*B levels in cells with or without CTRP3 were determined by western blot. High glucose-induced nuclear translocation of NF-*κ*B was inhibited by CTRP3 (Figure 2(d)). High glucose-triggered upregulation of p-NF-*κ*B in the cytoplasm was lower in cells treated with HG+CTRP3 compared with cells treated with HG alone (Figure 2(d)).

### 3.3. CTRP3 Provided Antiapoptosis Effect in HUVECs after HG Exposure

**Figure 3:**
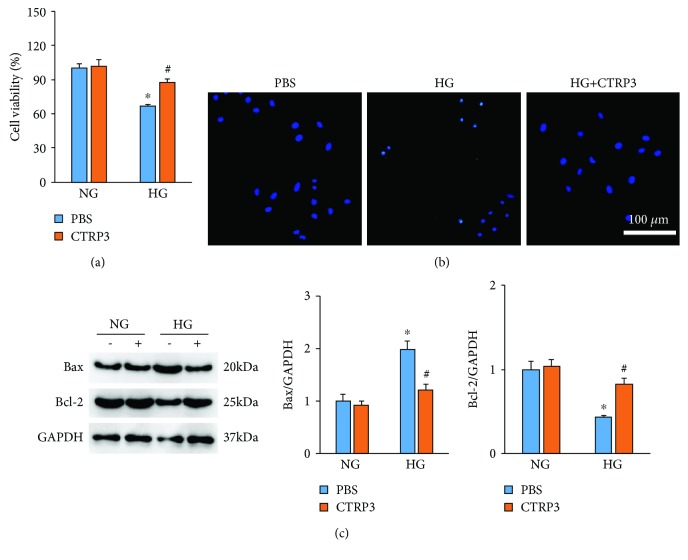
CTRP3 reduces the level of apoptosis in HUVECs induced by high glucose. (a) Cell viability in HUVECs induced by high glucose (*n* = 5). (b) Cell apoptosis as detected by TUNEL staining. (c) The protein expression of Bax and Bcl-2 was detected by western blot analysis (*n* = 5). Data are expressed as mean ± SD of five independent experiments. ^∗^*P* < 0.05 when compared to the NG group; ^#^*P* < 0.05 when compared to the HG group.

The data in our study indicated that compared with the NG group, HG treatment markedly decreased cell viability of HUVECs. However, CTRP3 treatment almost restored cell viability to the normal level (Figure 3(a)). As shown in Figure 3(b), 72 hours post HG exposure, an increased level of apoptosis was observed in HG-treated cells and CTRP3 administration could decrease the proportion of TUNEL-positive cells (Figure 3(b)). Western blot results showed that CTRP3 treatment upregulated Bcl-2 protein expression and downregulated Bax protein expression in HUVECs after HG exposure (Figure 3(c)). However, there was no change in these apoptosis-related proteins between the NG+PBS group and the NG+CTRP3 group (Figure 3(c)).

### 3.4. CTRP3 Could Activate AKT-mTOR Signaling Pathway in HUVECs after HG Exposure

**Figure 4:**
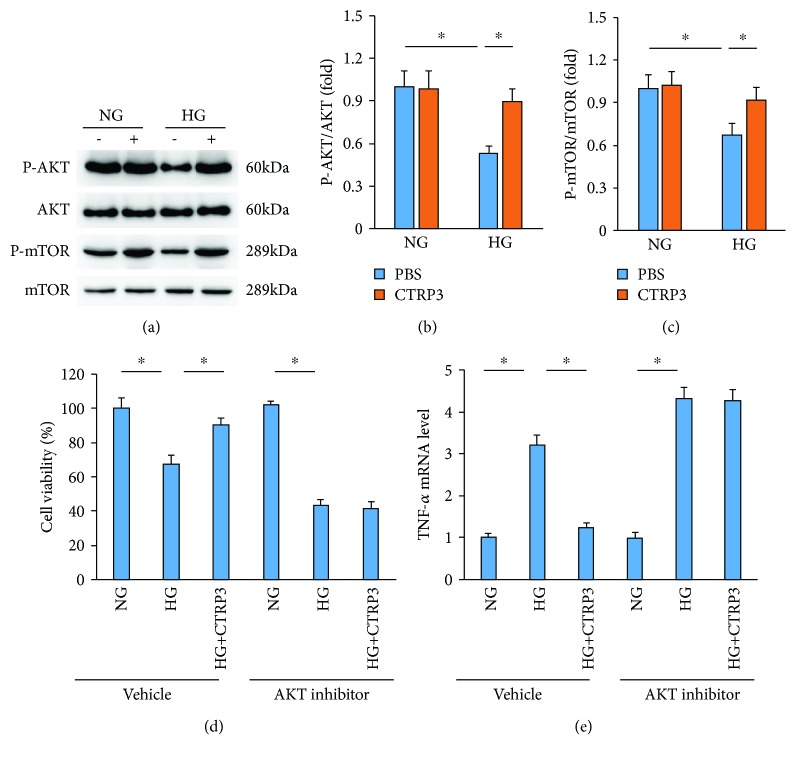
CTRP3 activated the AKT signaling pathway. (a–c) The protein expression of AKT and mTOR was detected by western blot analysis (*n* = 5). (d) Cell viability in HUVECs induced by high glucose (*n* = 5). (e) The mRNA expression of TNF-*α* in HUVECs exposed to high glucose (*n* = 5). Data are expressed as mean ± SD of five independent experiments. ^∗^*P* < 0.05 when compared to the matched control.

To explore the mechanism by which CTRP3 inhibited inflammation and cell apoptosis, we first detected the alteration in AKT-mTOR signaling pathway in HUVECs after HG exposure. There was no difference in the phosphorylation levels of AKT and mTOR between the NG+PBS and NG+CTRP3 groups (Figures 4(a)–4(c)). HG exposure markedly decreased the phosphorylation levels of AKT-mTOR in HUVECs; however, CTRP3 treatment restores the phosphorylation levels of AKT-mTOR (Figures 4(a)–4(c)). Next, we examined whether the activation of AKT-mTOR signaling pathway was involved in CTRP3-provided protection. The data in our study demonstrated that the AKT inhibitor was capable of blocking the protective effects of CTRP3 on cell viability and inflammation in HUVECs after HG exposure (Figures 4(d) and 4(e)).

### 3.5. CTRP3 Deletion Increased the Level of TNF-*α* and Decreased Cell Viability at Baseline

**Figure 5:**
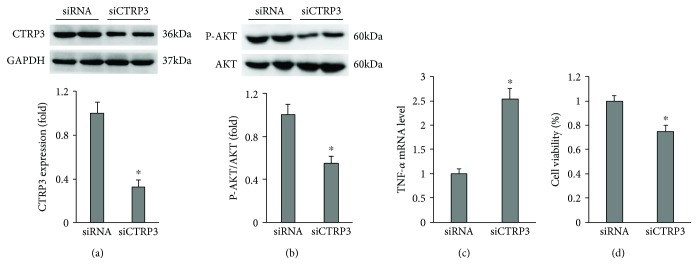
CTRP3 deficiency increased inflammation and cell death at baseline. (a) Protein level of CTRP3 (*n* = 5). (b) The protein expression of AKT was detected by western blot analysis (*n* = 5). (c) The mRNA expression of inflammatory factor in HUVECs (*n* = 5). (d) Cell viability in HUVECs (*n* = 5). Data are expressed as mean ± SD of five independent experiments. ^∗^*P* < 0.05 when compared to the matched control.

Next, we investigated whether the endogenous CTRP3 deficiency affected HUVECs. We knocked down CTRP3 in HUVECs. Downregulation of CTRP3, confirmed by western blot analysis (Figure 5(a)), decreased the phosphorylation levels of AKT in HUVECs (Figure 5(b)). CTRP3 deficiency increased the mRNA level of TNF-*α* and decreased the viability of HUVECs in basal conditions (Figures 5(c) and 5(d)).

## 4. Discussion

In this study, our results demonstrated that CTRP3 mRNA and protein expression were significantly decreased in HG-treated HUVECs. Using recombinant human CTRP3 globular form, we found that CTRP3 administration inhibited HG exposure-induced inflammatory factor accumulation and cell loss in HUVECs. Mechanistically, CTRP3 could activate the AKT-mTOR signaling pathway in HUVECs, and AKT inhibition could abolish the protective effects provided by CTRP3 treatment. Knockdown of CTRP3 resulted in inflammation accumulation and cell death in vitro.

CTRP3 is an adipokine associated with various cardiovascular diseases, including diabetic cardiomyopathy, agent-related cardiac injury, and cardiac hypertrophy [9, 11, 16]. The data in our study found that CTRP3 mRNA and protein expression were downregulated in the cells treated with HG, which was in line with the expression of CTRP3 in animals with myocardial ischemia and diabetes [12, 17]. CTRP3 was also found to be decreased in obesity-related male reproductive dysfunction in mice [18]. However, there exists an opposite voice that CTRP3 mRNA and protein were increased in hypertrophic hearts [16]. The discrepancy between these studies might be explained by the different animal diseases and different pathological conditions.

The alteration of CTRP3 expression implies that CTRP3 might play a role in high glucose-induced endothelial dysfunction. As expected, CTRP3 treatment suppressed HG-induced inflammation accumulation and endothelial apoptosis in vitro. Our finding was in agreement with previous studies which described protective roles in cardiometabolic diseases [11, 12, 18]. However, Ma et al. found that CTRP3 overexpression promoted pressure overload which caused hypertrophic response in mice [16]. These incompatible results may be explained by the different intervention approaches.

The microinflammatory state is closely associated with the development of diabetes-related endothelial dysfunction [19]. High glucose could result in the excessive production of TNF-*α* and IL-1*β*, which caused the activation of NF-*κ*B and led to amplification of inflammatory response [20]. Hyperglycemia also directly triggered the activation of NF-*κ*B [21]. After that, NF-*κ*B translocated into the nucleus and then regulated the expression of MCP-1 [22]. Forced expression of a dominant negative mutant of NF-*κ*B inhibited proinflammatory gene expression in endothelial cells [23]. Our study found that CTRP3 treatment reduced the mRNA levels of TNF-*α*, IL-1*β*, IL-6, and MCP-1 in HUVECs after HG exposure. Sustained inflammation resulted in the apoptosis of the endothelium. Therefore, we detected the effect of CTRP3 on HG-induced endothelial apoptosis and found that CTRP3 also improved cell viability and reduced cell apoptosis in HG-treated HUVECs.

The AKT-mTOR signaling pathway was closely related with the development and progression of high glucose-induced endothelial dysfunction [24, 25]. AKT, a serine/threonine protein kinase, is known as a focal point for signal transduction pathways responsible for cell survival [26]. AKT activation could promote cell viability by blocking Bax conformational change [27]. Therefore, we first detected the alteration of AKT in HG-treated HUVECs and found that phosphorylation levels of AKT and mTOR were significantly decreased after HG treatment. However, CTRP3 restored AKT phosphorylation to the normal level, which was consistent with a previous study [9]. In addition, we also found that CTRP3 reduced Bax expression and increased Bcl-2 expression in HUVECs. Ma et al. found that CTRP3 exerted its protection via AMP-activated protein kinase *α* (AMPK*α*) not AKT signaling pathway [9]. To verify the hypothesis that CTRP3 provided protection against endothelial dysfunction via the activation of AKT signaling pathway, we used an AKT inhibitor. CTRP3 lost its protection against cell loss and inflammation after AKT inhibition, implying that CTRP3 attenuated HG-induced inflammation and apoptosis through the activation of the AKT-mTOR signaling pathway.

Collectively, the present study demonstrated that CTRP3 improves cell viability and ameliorates cell inflammation via activating the AKT pathway. Our study provides evidence for the application of CTRP3 in the treatment of high glucose-induced endothelial dysfunction.

## Data Availability

The data that support the findings of this study are available from the corresponding authors upon reasonable request.
